# Supplementary figures and images for: Variant-specific priors clarify colocalisation analysis
Source: PLoS Genet. 2025 May 27;21(5):e1011697. doi: 10.1371/journal.pgen.1011697 (PMC12140431; doi:10.1371/journal.pgen.1011697)

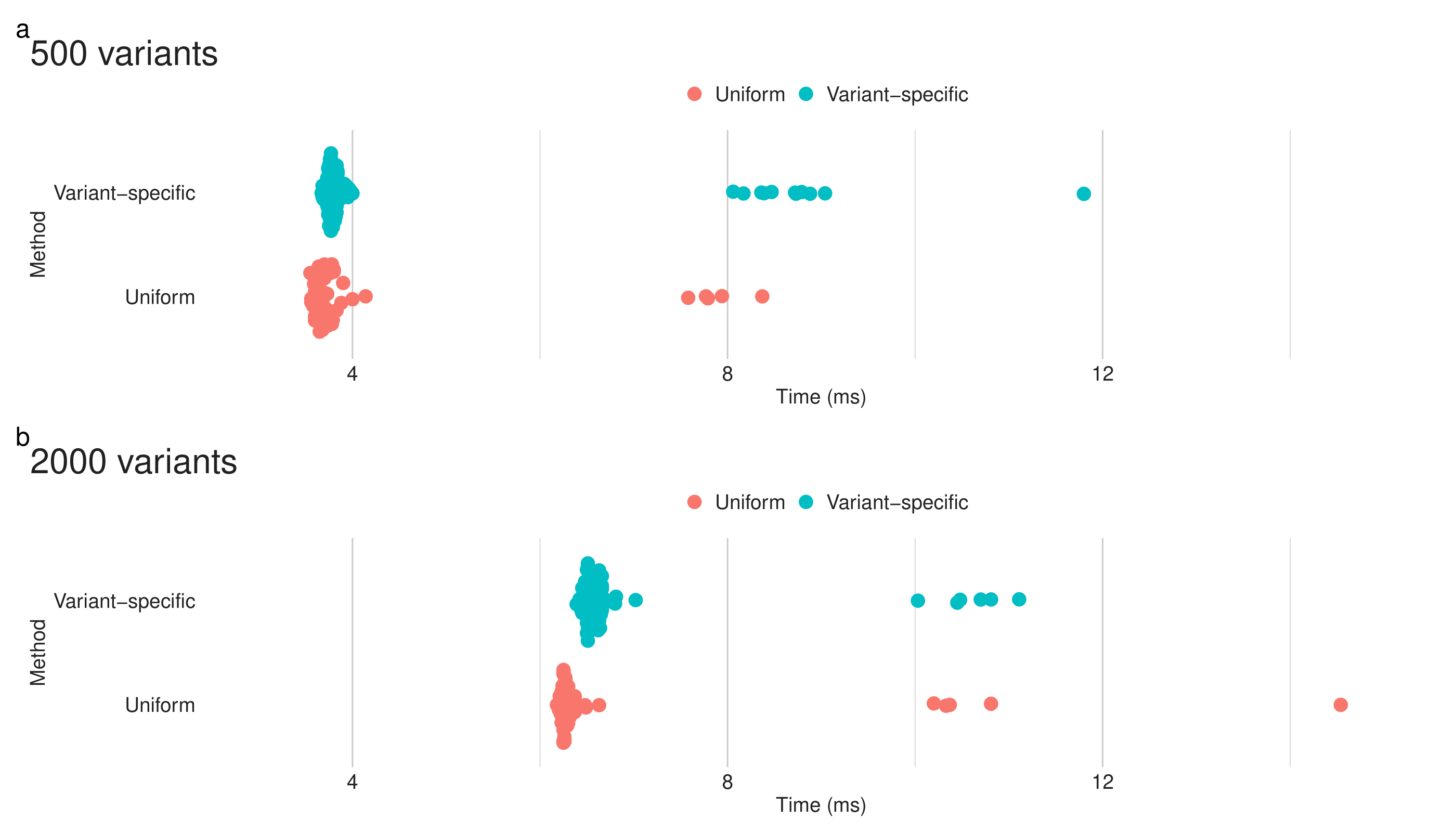

Supplement: S1 Fig — Beeswarm plot of execution time of coloc with variant-specific and uniform priors. (a) Datasets with 500 variants. (b) Datasets with 2000 variants. Using variant-specific priors has no meaningful effect on speed, especially when the number of variants is small. (TIFF) [file pgen.1011697.s002.tiff]

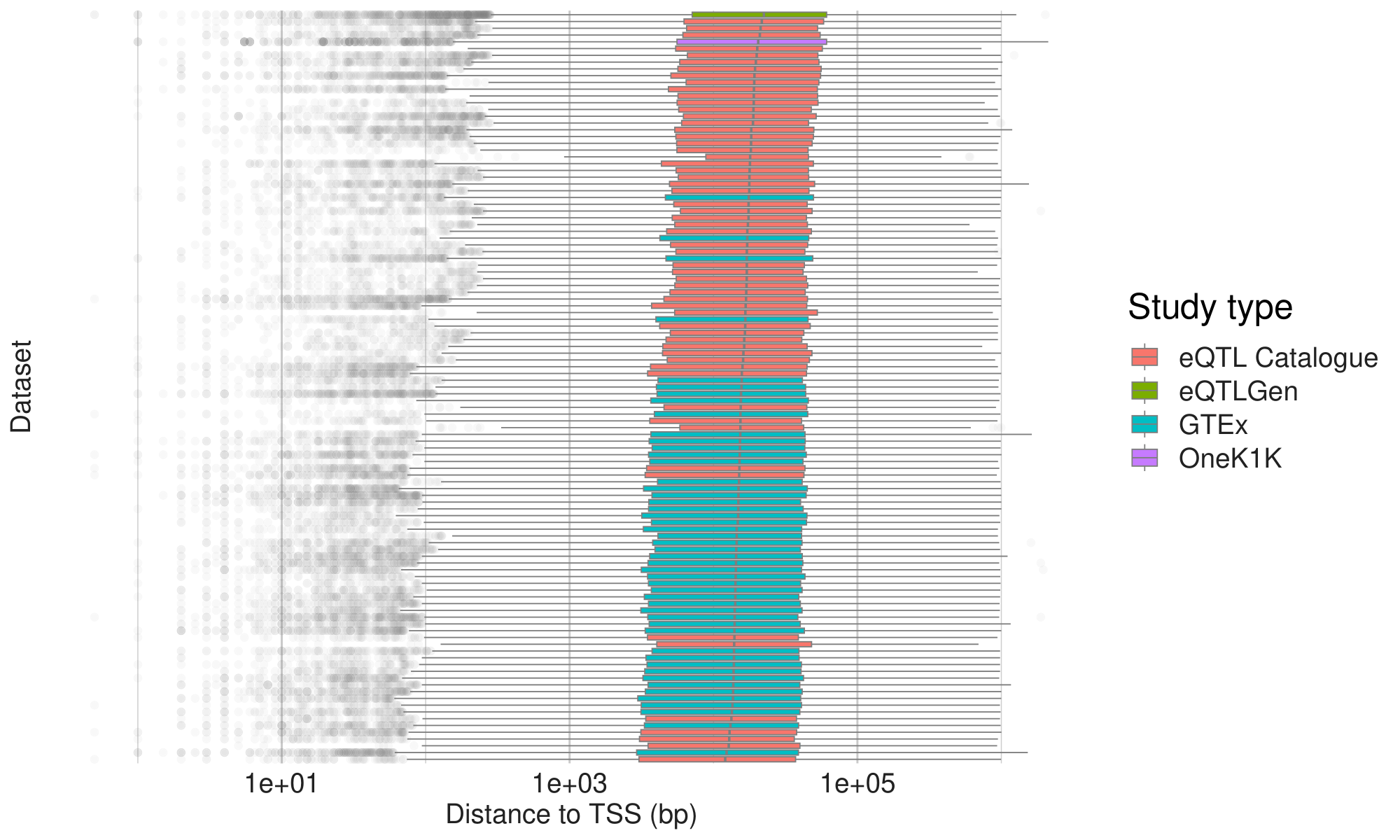

Supplement: S2 Fig — Boxplots of distance (measured in base pairs, log10 scale) of eQTLs to TSS for genome-wide significant eQTLs in v6 of the eQTL catalogue (including GTEx v8), eQTLGen and OneK1K dataset. Each boxplot corresponds to a different study. (TIFF) [file pgen.1011697.s003.tiff]

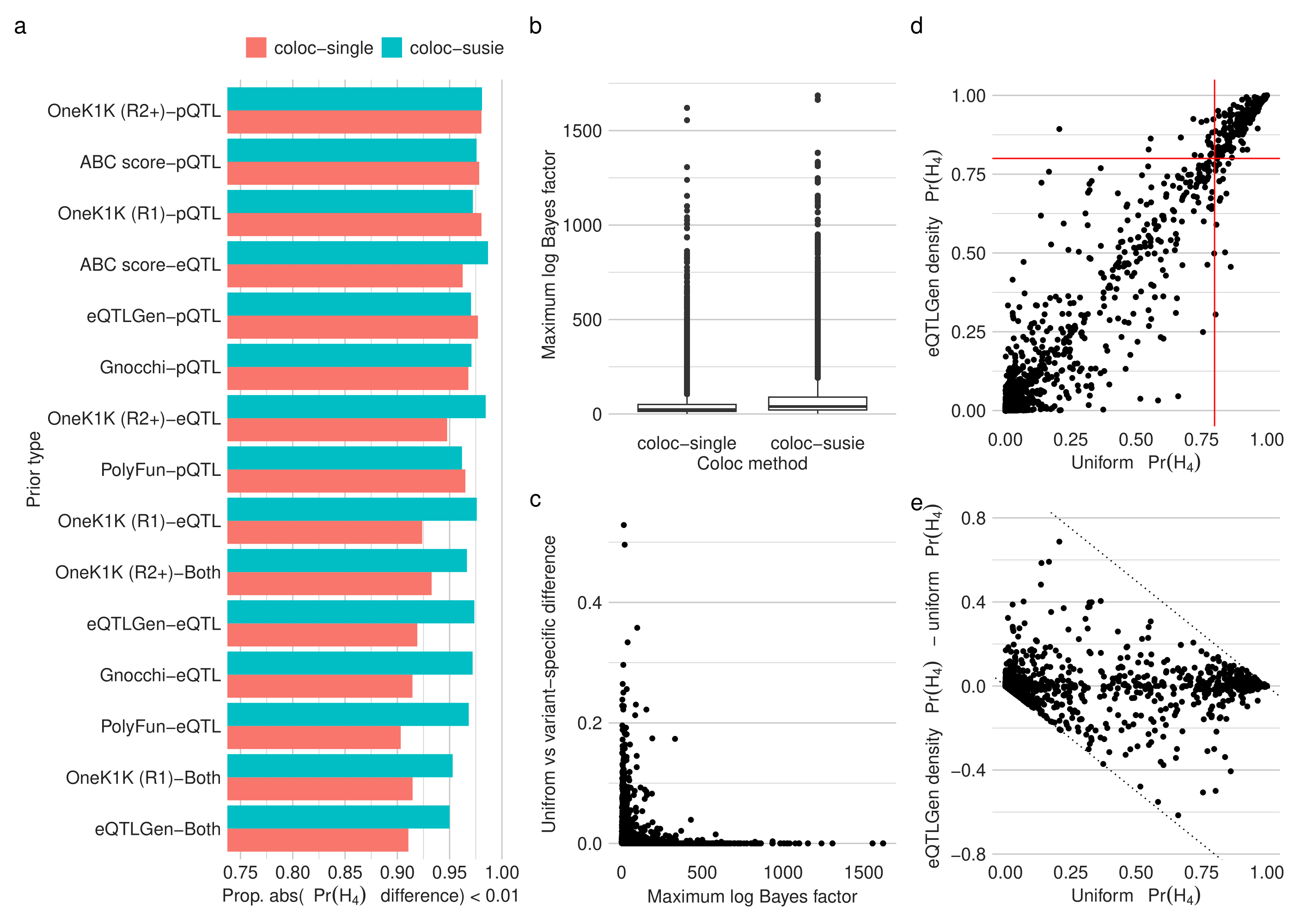

Supplement: S5 Fig — (a) Plot of the proportion of loci with absolute value change in Pr(H4) less than 0.01 for coloc method with all priors. (b) Boxplot of maximum log Bayes factor calculated by coloc-single or calculated by SuSiE and used as input to coloc-susie across loci. (c) Scatter plot of maximum log Bayes factor for coloc-single method against the difference in uniform and variant-specific priors, taking the median over prior information sources. Each point is a locus. (d) Scatter plot of Pr(H4) calculated with a uniform prior vs with an eQTLGen-estimated eQTL-TSS density prior across all tested pQTL-eQTL loci for which colocalisation was performed. The red lines show the 0.8 significance threshold for both coloc with uniform and variant-specific priors. (e) Scatter plot of Pr(H4) with uniform priors vs the difference between Pr(H4) with uniform and eQTLGen-estimated eQTL-TSS density prior across all pQTL-eQTL loci where colocalisation was performed. The grey lines show the maximum possible values of the difference in colocalisation probabilities given the value of Pr(H4) calculated with uniform prior probabilities. (TIFF) [file pgen.1011697.s006.tiff]

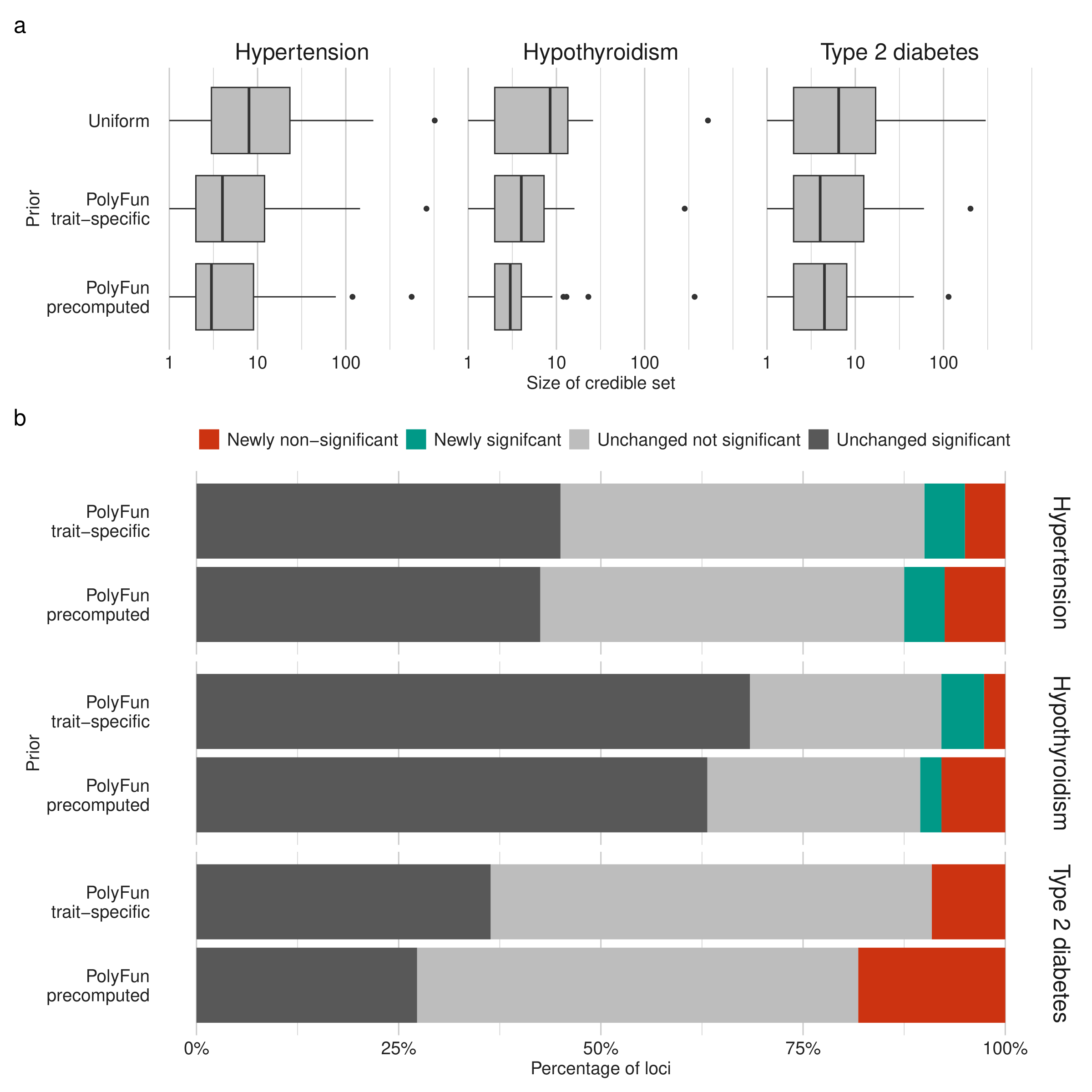

Supplement: S7 Fig — (a) Credible set size using different priors across three traits. (b) As in ??, the effect of priors on colocalisation significance across three UK Biobank traits. Here we also consider applying the PolyFun priors to the GWAS dataset and eQTLGen prior to eQTLGen dataset simultaneously. (TIFF) [file pgen.1011697.s008.tiff]
