# Supplementary material for: Variant-specific priors clarify colocalisation analysis
Source: PLoS Genet. 2025 May 27;21(5):e1011697. doi: 10.1371/journal.pgen.1011697 (PMC12140431; doi:10.1371/journal.pgen.1011697)
Supplement: Attachment — Submitted filename: vsp-reviewer-response.pdf [file pgen.1011697.s011.pdf]

Reviewer #1:

Summary of the Paper:

The manuscript addresses the challenge of linking GWAS variants to their causal genes using the coloc method, a widely used approach for statistical colocalisation analysis. The authors propose incorporating variant-specific prior probabilities, based on diverse data sources such as PolyFun, non-coding constraint (Gnocchi), and enhancer-gene link scores (ABC), into coloc to improve performance. Their findings show that using these priors improves colocalisation accuracy in up to 13.5% of loci, demonstrating the benefits of integrating additional variant-level information.

Scientific Novelty:

The study is novel in extending the coloc method by introducing variant-specific prior probabilities to account for the heterogeneity of variant information, a concept well established in GWAS fine-mapping but not previously explored in colocalisation. This work bridges the gap between fine-mapping and colocalisation analysis, addressing the limitations of uniform priors in coloc. The systematic evaluation of multiple sources of variant information in real-world pQTL-eQTL and GWAS-eQTL colocalisation adds to the value of this contribution.

Major Concerns:

1. Limited improvement in both simulation and real data scenarios: Despite the potential of variant-specific priors, the reported improvement in performance (2-3% increase in recall and precision) is modest. The study highlights that the impact of these priors was often small, especially in GWAS-eQTL colocalisation. This may question the practical utility of the method for large-scale applications, where computational costs must be justified by substantial performance gains.

We agree that relative performance is more modest than we had anticipated. We believe this is in itself an important finding - methods which incorporate variable priors in fine mapping have shown substantial improvements, so observing this did not translate to coloc prompted us to think carefully about the links between fine-mapping and coloc. We concluded that because most coloc conclusions are already made with high confidence (i.e. posterior probabilities are concentrated around 0 or 1), there is not much space for the additional information provided by the prior to change the posterior. However, given the widespread use of coloc, we feel that improvements in performance in the minority of cases where conclusions are of moderate confidence have the potential to be beneficial.

We also agree that considering trade-offs between method complexity and computational performance is important. We note that in this case, due to the analytic nature of coloc's calculations and the decision to require pre-calculated variant-specific priors, there is practically

no added computational cost. We have updated the main text and added a microbenchmark of coloc with and without variant-specific priors, to clarify this point. The relevant text is:

"Using variant-specific priors is not meaningfully slower than standard coloc (Supplementary Figure 1)

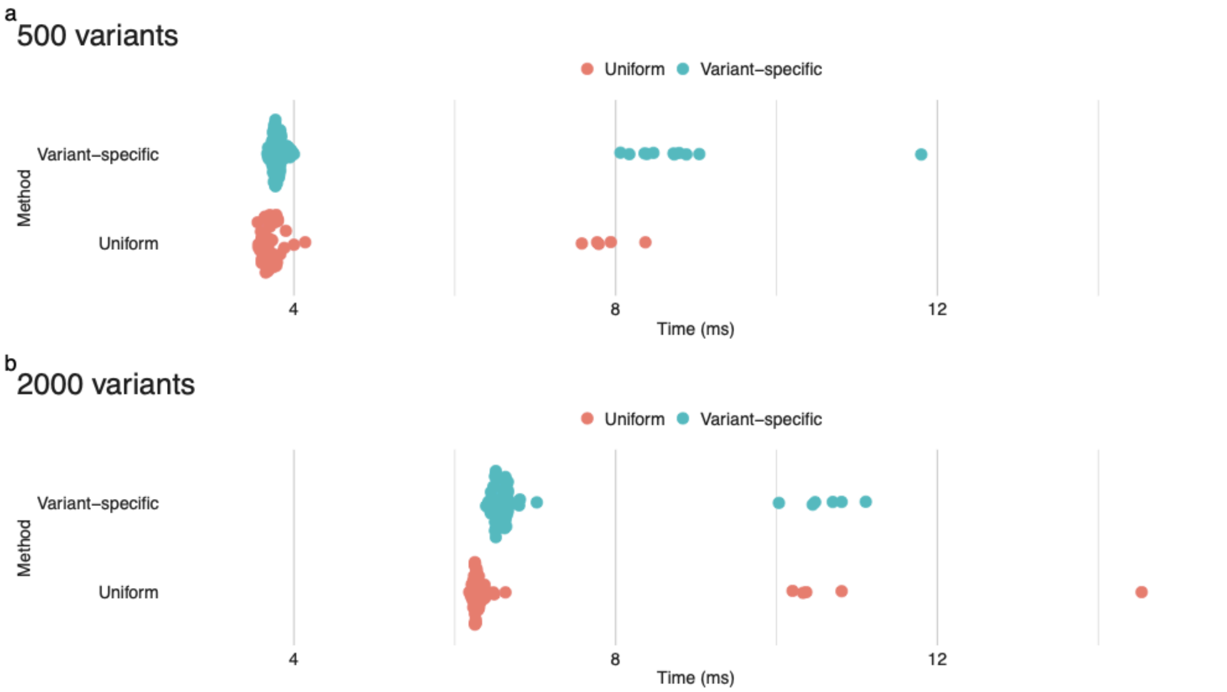

Supplementary Figure 1: Benchmark of coloc execution speed with variant-specific and uniform priors *Beeswarm plot of execution time of coloc with variant-specific and uniform priors. a) Datasets with 500 variants. b) Datasets with 2000 variants. Using variant-specific priors has no meaningful effect on speed, especially when the number of variants is small. See Methods for details.*

2. Lack of technical contribution: The author did not make attempt to improve the co-localization fine-mapping performance. For instance, the importance of the annotations may change depending on the traits. In PolyFun and SparsePro, the annotation weights are learned as *empirical priors*. But in the proposed approach the prior (while variant-specific) are fixed.

We thank the reviewer for highlighting this important aspect of our approach. We chose specifically to use fixed prior probabilities for two reasons. First, this approach ensures that coloc using variant-specific priors is not slower than standard coloc. (We have now included a benchmark in the paper that shows this point). Second, the approach is modular in the sense that it allows the user to select or manually specify any prior weights, exploiting the ever growing amounts of information about genetic variants and gene regulatory architecture. Users can also use methods, such as PolyFun, which can define priors taking into account multiple annotations and with respect to particular traits. We have now added the following text to the results section

to make both of these considerations more explicit. In addition, we now examine the use of trait-specific PolyFun computed prior probabilities in the manuscript, in response to the reviewer's point 3 below.

"In addition, for consistency we added the ability to use priors weights in the finemap.abf() function. Users of coloc can supply any weights they think are informative for the causality of variants a priori*, including the methods of specifying weights we assess in this manuscript, information about variant level properties in trait relevant contexts, such as single-cell enhancer-gene maps (22), and priors that have been empirically estimated for their trait of interest, for example using PolyFun. As coloc does not estimate variant-specific priors, using variant-specific priors is not meaningfully slower than standard coloc (Supplementary Figure 1). For mathematical details of the implementation of variant-specific priors see Methods."*

3. Lack of direct application of PolyFun to the GWAS traits of interest: The authors mention that, for computational reasons, PolyFun was not directly applied to the GWAS datasets analyzed, and pre-computed priors from UK Biobank traits were used instead. Given that PolyFun is dataset-specific, this compromises the applicability of these results to different traits, and the conclusions drawn may not fully reflect the method's potential. Another alternative is SparsePro (Zhang et al., PloS Genetics 2024) and SharePro (Zhang et al., Bioinformatics 2024), which was published more recently and more efficient than PolyFun.

We have now run PolyFun on traits drawn from the UK Biobank, to produce trait specific prior probabilities. We observed that these trait-specific probabilities were visually similar to the precomputed prior probabilities. In addition, the trait-specific probabilities had a smaller effect, both on credible set size and colocalization significance, than precomputed PolyFun prior. We could only run PolyFun on traits in the UK Biobank due the computationally intensive nature of PolyFun and the need for functional annotation and LD data not readily available outside the UK Biobank. Based on these observations we conclude that trait-specific PolyFun priors are unlikely to outperform precomputed priors for colocalization analysis, and are almost certainly not worth the additional complexity of computing them. We have included the following text and figures in the paper to convey these findings.

"Given the strong reported performance of PolyFun for fine-mapping we also investigated whether PolyFun had a larger impact on colocalisation significance when its prior probabilities were estimated in a trait-specific manner. We estimated trait-specific prior probabilities for three traits in the UK Biobank - Type 2 diabetes, Hypertension and Hypothyroidism (see Methods for details). We did not use trait-specific priors in the pQTL-eQTL comparison or analysis of FinnGen GWAS traits as the prior estimation is very computationally demanding and requires additional functional annotation and LD information which is not readily accessible outside the UK Biobank. First, visualising the priors in the same region as Figure 1 (Figure S6), showed that the trait-specific priors had a broadly similar form to the precomputed priors. Next, we assessed the impact of the precomputed and traits-specific priors on credible set size (Figure S7a). As expected, the PolyFun priors were able to decrease the credible set size compared to the uniform prior. However, we observed that the precomputed priors generally had a larger impact

than the trait-specific priors. Finally, we performed colocalisation analysis between the three traits and five eQTL datasets in disease relevant tissues (Table S3, see Methods for details). In this analysis we again found that the trait-specific priors had less impact than the precomputed prior on colocalisation significance (Figure S7b). Taken together, these observations suggest that trait-specific PolyFun priors are unlikely to outperform the precomputed priors, cautioning against their use, especially given the large computational cost of estimating the trait-specific priors."

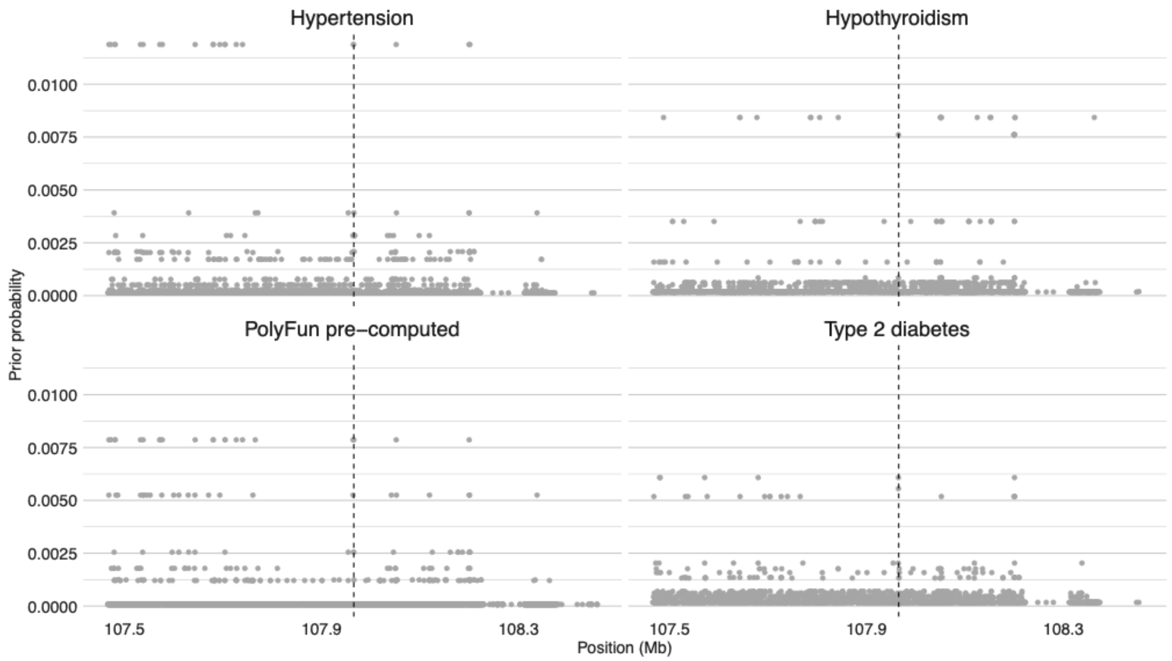

Supplementary Figure 6: Trait-specific prior and precomputed PolyFun calculated prior probabilities. *The probabilities are displayed in a +/-500kB region around the canonical TSS, shown by the dashed black line, of the VAV3 gene; the same region as in Figure 1*

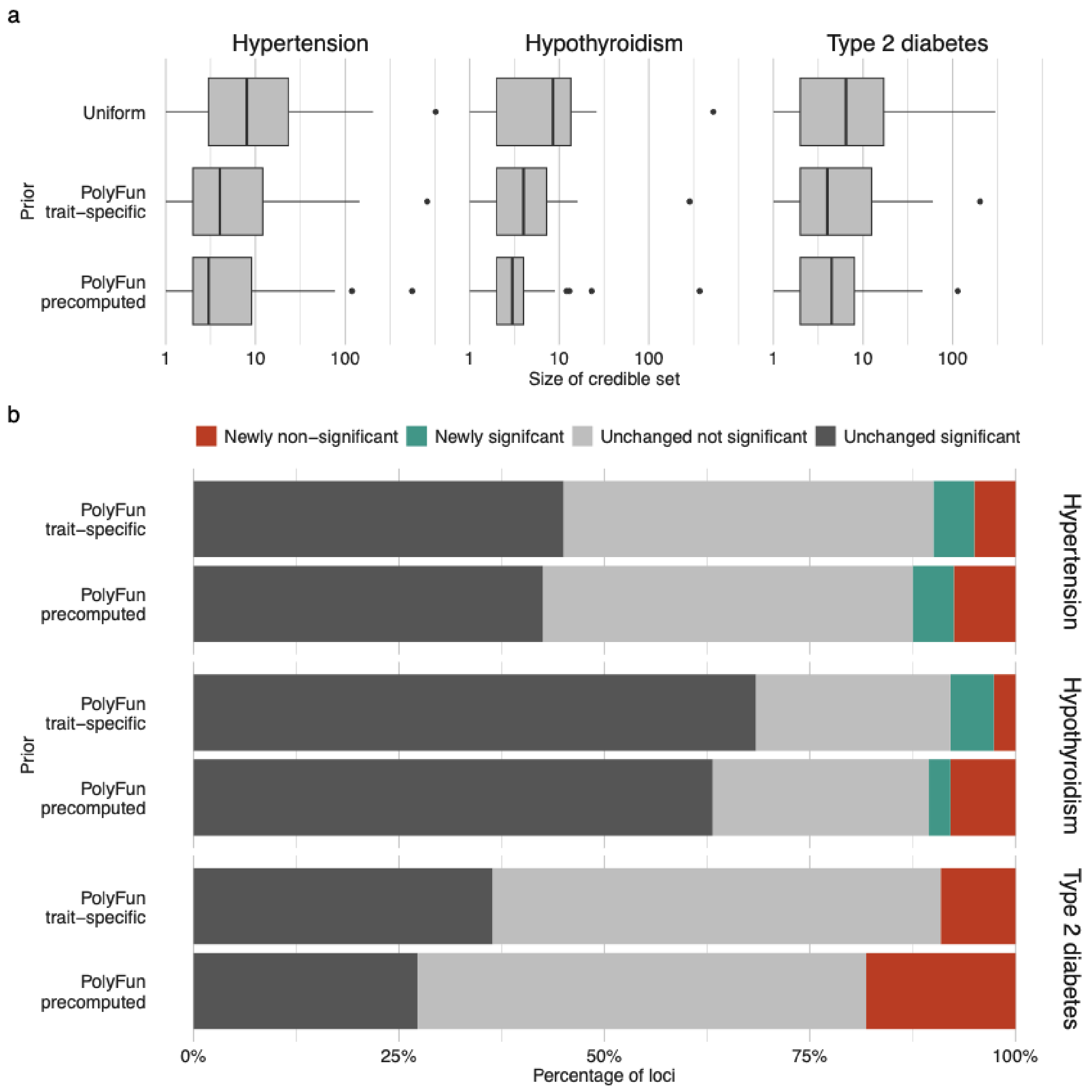

Supplementary Figure 7: The impact of PolyFun and eQTLGen priors in the UK Biobank trait. a) Credible set size using different priors across three traits. b) As in Figure 4, the effect of priors on colocalisation significance across three UK Biobank traits using the PolyFun trait-specific and precomputed priors applied to the GWAS dataset.

4. Generalizability to other GWAS contexts: The study largely focuses on pQTL-eQTL colocalisation with minimal testing on diverse GWAS traits. The results of colocalisation in autoimmune disease, type 2 diabetes, and hypertension in FinnGen are briefly mentioned, but

the manuscript would benefit from further exploration and a broader comparison to other trait categories.

We have now extended the GWAS analysis to include seven other GWAS traits, drawn from FinnGen, taking the total number of traits to ten and greatly diversifying the types of traits we assess. The new traits include two quantitative traits (height and weight) and three traits with lower case numbers (IBD, type 1 diabetes and rheumatoid arthritis). Overall, when adding more traits we found concordant results with the original analysis. In most traits, 5-12% of colocalisations changed significance when using variant-specific priors. Across traits, colocalization methods and priors, single-causal variant coloc using the eQTLGen priors applied to height had the highest proportion of colocalizations with changed significance at 14%. We have updated Figure 4 to reflect the changes and added the following text. While revising the figure, we also changed it to display the proportion of loci rather than the number, which better visualises changes across traits with widely varying numbers of loci. The relevant updated text and figure are:

“Across trait-dataset pairs the variant-specific priors had a moderate impact on changing whether a variant-gene pair was significant (Figure 4a), with at most 14\% of colocalisations with some evidence of colocalisations (\Pr(H_4) > 0.5)) changing significance at the 0.8 threshold. For coloc with the single causal variant assumption, height, hypertension and IBD had the highest proportion of colocalisations change significance while for coloc-susie weight and Type 1 diabetes had the highest proportion. Notably, across priors and colocalisation methods no colocalisations for rheumatoid arthritis changed significance.”

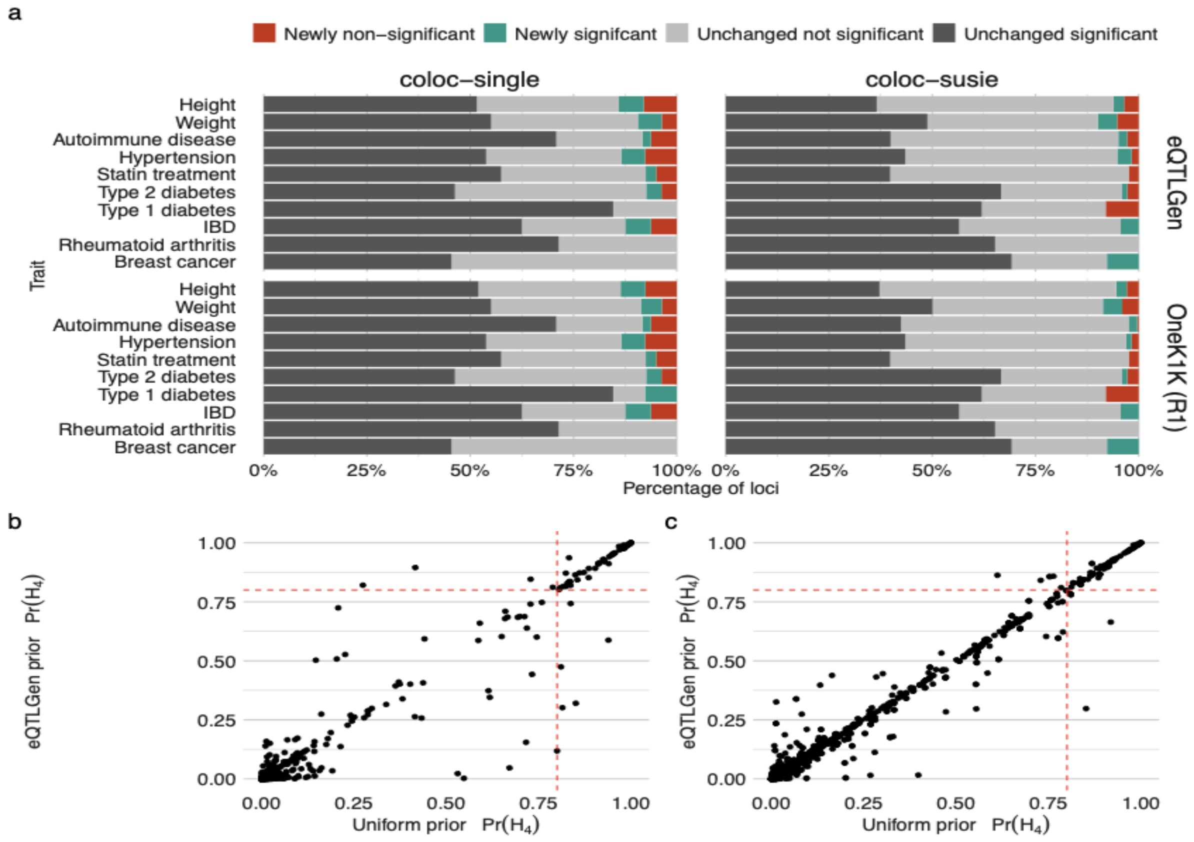

Figure 4: GWAS-eQTL colocalisation analysis *a) Number of loci with some evidence of colocalisation ($\Pr(H_4) > 0.5$) using a uniform prior coloured by the effect of a variant-specific priors on colocalisation significance threshold of 0.8. Results are for the eQTLGen and OneK1K round 1-estimated priors and coloc using SuSiE (coloc-susie) and coloc with the single variant assumption (coloc-single). Unlike in the pQTL-eQTL analysis, here colocalisations with each credible set are counted as separate colocalisations. b) Scatter plot of $\Pr(H_4)$ computed using uniform and eQTLGen eQTL-TSS distance density priors for coloc with the single causal variant assumption applied to Autoimmune disease trait colocalisations c) As in b) but using coloc with SuSiE.*

5. Absence of strong benchmarks for comparison: While the study uses pQTL-eQTL colocalisation as a ground truth benchmark, it remains unclear how the method compares against other cutting-edge colocalisation methods (e.g., SharePro) or how well it would perform in a setting with more complex polygenic traits. Introducing stronger benchmarks would improve the robustness of the results.

There is now a baseline benchmark comparing coloc, SharePro and other colocalisation methods (https://www.biorxiv.org/content/10.1101/2024.10.14.617627v1), which is now referenced in the manuscript. (See relevant text below). However, the focus of our manuscript is how to include additional information through variant-specific priors and whether this can

improve colocalisation performance. No other methods, including SharePro, offer this option, so it is not possible to benchmark our novel implementation against that of other colocalisation approaches. Our simulation results show that our implementation is working as intended, in both H4 and H3 simulation settings.

"Different statistical methods have been developed to assess colocalisation, including SharePro, PWCoCo and coloc (7, 17, 18). (See (19) for a recent benchmark of Bayesian colocalisation methods)."

Minor Concerns:

1. Code and reproducibility: The paper references that the code for implementing the method is available in a GitHub repository. It would be beneficial to explicitly include a link in the manuscript and provide a minimal working example to allow users to apply the methods on their own data.

We have now included links in the "Code availability" section of the manuscript to:

- a GitHub repository containing code to reproduce the analysis in the manuscript;
- the branch of the coloc repository containing the implementation of variant-specific priors, and;
- a new vignette in the coloc repository that contains information about how to use variant-specific priors in practice (https://github.com/chr1swallace/coloc/blob/variant-specific-priors/vignettes/a07_variant-specific-priors.Rmd)

2. The first two sections under Results are mostly method description would be more appropriate under Methods section

We have now greatly shortened these sections, moving the bulk of their content to Methods. The first subsection describing the implementation in coloc now also highlights two facts that were not sufficiently highlighted in previous version of the manuscript: that users can supply any variant-specific prior probabilities and that using variant specific priors incurs no extra computational cost. The relevant revised text at the start of the Methods section is:

"Incorporating variant-specific prior probabilities in coloc

We implemented the ability to specify variant-specific priors in the widely used coloc package for statistical colocalisation analysis (Figure 1b). The coloc.abf(), coloc.susie() and coloc.bf_bf() functions in coloc have been updated to include two new arguments: \texttt{prior_weights1} and prior_weights2, which specify non-negative weights for the probability of a variant being causal for trait 1 and trait 2 respectively. In addition, for consistency we added the ability to use priors weights in the finemap.abf() function. Users of coloc can supply any weights they think are informative for the causality of variants a priori, including the methods of specifying weights we assess in this manuscript, information about variant level properties in trait relevant contexts,

such as single-cell enhancer-gene maps (22), and priors that have been empirically estimated for their trait of interest, for example using PolyFun. Using variant-specific priors is not slower than standard coloc (Figure S1). For mathematical details of the implementation of variant-specific priors see Methods.

Specification of variant-specific prior probabilities

Various methods are available for specifying variant-specific weights. In this paper we assessed four types of sources for specifying variant-specific weights. Three sources were drawn from public datasets: the output of the PolyFun method (13), `Gnocchi' score, a measure of non-coding genomic constraint (11) and genome-wide activity-by-contact enhancer-gene link scores (10, 23). For these sources we accessed publicly available data and performed minor processing, mainly to account for SNPs which did not have specific prior values (for further details see Methods). We sought to use empirical eQTL-TSS distance densities as an additional source of prior information. We estimated densities from all significant eQTLs in eQTLGen (24) and both significant round 1 eQTLs and significant round 2-5 conditional eQTLSs OneK1K dataset (25) after finding that the estimated densities were not highly tissues specific (Figure S2) and that these datasets had more distal eQTLs relative to the TSS compared to other datasets (See Methods for details). Overall, we used 6 sources of variant-specific prior probabilities (Table 1), shown in Figure 1a for a +-500$Kb window around the TSS of VAV3. The eQTL-TSS density priors place most mass in a small region around the TSS, with the OneK1K round 2+ estimated prior being less concentrated around the TSS. The ABC score-prior is also centred around the TSS, but is less smooth."

3. Threshold choices: The manuscript uses a threshold of Pr(H4) > 0.8 to define colocalisation significance. It would be useful to provide additional context or references for why this threshold was chosen, particularly since other colocalisation studies may use different thresholds.

We thank the reviewers for raising this important point. We have added a section to the Methods, referenced at the first use of the Pr(H4 > 0.8) threshold and reproduced below, which expands on the chosen threshold. We also note that the receiver-operator curve analysis of the pQTL-eQTL benchmark is designed to assess the method at a range (0.5-0.95) of plausible colocalisation thresholds.

"In this paper, we use the threshold of Pr(H4) > 0.8 as the standard threshold for calling a colocalisation as significant. The threshold was first used in the original paper describing coloc, where colocalisation with >80\ (i.e. >0.8) Pr(H4) > 0.8 are referred to as `positive colocalisation results' (7). Today, the threshold is widely used to call colocalisations as significant in applied papers that use coloc (see, for example, (28, 38, 39). Additionally, an empirical attempt to evaluate this threshold concluded that both coloc H4 > 0.8 and GWAS p < 5 x 10-8 achieved similar performance in predicting validation in larger GWAS from results of a smaller GWAS. Finally, other colocalisation methods such as SharePro also use the 0.8 threshold (18)"

4. It would be helpful to have an overview Figure as Figure 1 to give a high-level conceptual diagram of the proposed method.

We agree, and have updated the figure to both display the prior probabilities and give an overview of the methodological contribution of the paper. The updated figure (Figure 1) is below. (In an earlier version of the figure the coloc logo was used in place of the word coloc but the PLOS Genetics team indicated that they are unable to permit the use of a logo, even with permission.)

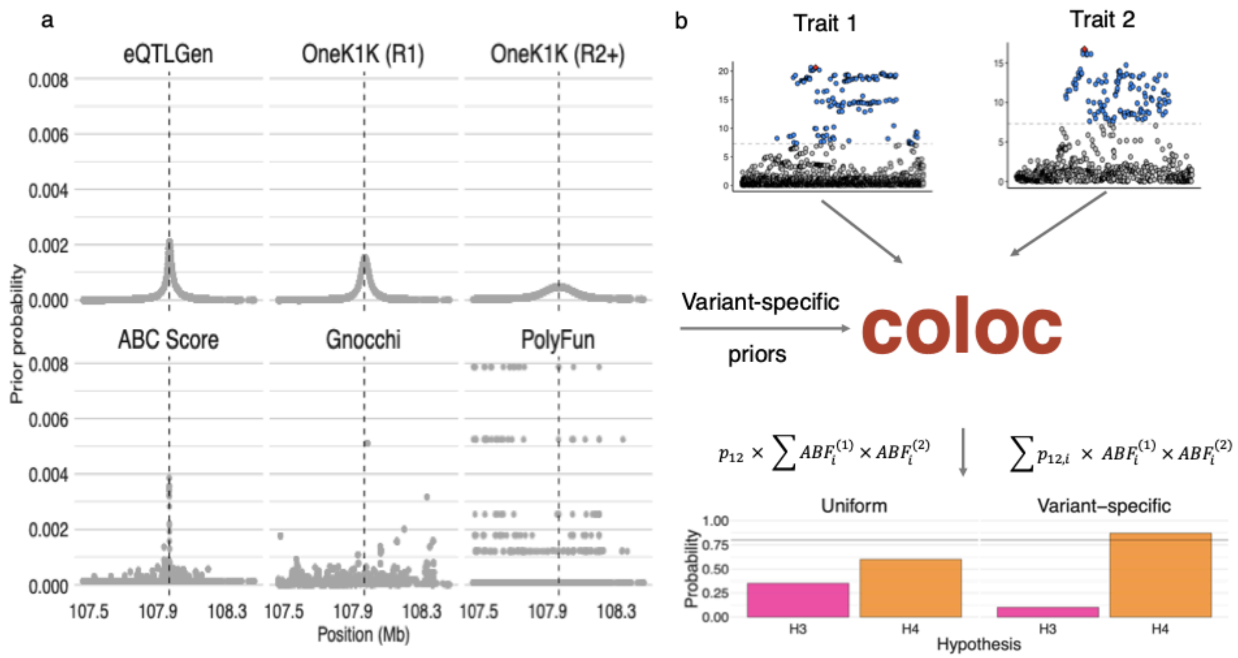

Figure 1: Example prior probabilities of causality and conceptual figure. *a) Prior probabilities from the six sources of prior probabilities: eQTLGen estimated eQTL-TSS distance density, OneK1K round 1 estimated eQTL-TSS distance density, OneK1K round 2+ estimated eQTL-TSS distance density, genome-wide enhancer-gene link scores calculated using the ABC score, Gnocchi non-coding constraint score and previously calculated PolyFun prior probabilities. The probabilities are displayed in a +/-500kB region around the canonical TSS, shown by the dashed black line, of the VAV3 genes b) A conceptual overview of coloc with variant-specific priors. The coloc method takes summary statistic for two traits and test colocalisation, now with the ability to all specify variant specific priors. The formulas shown are proportional to Pr(H4) using either variant-specific or uniform priors.}*

5. Supplementary material integration: There is heavy reliance on supplementary material for important methodological details. Integrating more of this content into the main manuscript, or at least summarizing key points, would improve readability and allow for a more thorough understanding without requiring extensive cross-referencing.

We thank the reviewer for raising this point. We have now expanded the section describing the implementation of variant-specific coloc in Methods (reproduced below), incorporating both material taken from the results section, following the reviewer's comment above, and the section justifying the properties of the approach we have moved from the supplementary material. We reproduce this section below. The only extra details now present in the Supplementary Material are a brief overview of the methodology of coloc and a discussion of the effect of specifying priors on both traits.

"The coloc.abf(), coloc.susie() and coloc.bf_bf() functions in coloc have been updated to include two new arguments: prior_weights1 and prior_weights2, which specify non-negative weights for the probability of a variant being causal for trait 1 and trait 2 respectively. These weights are used to calculated variant-specific prior probabilities, according to the formula

\begin{equation}

p_{k, i} = Q \times p_k \times \frac{w_{k, i}}{\sum_{i = 1}^Q w_{k, i}}, \ k \in \{1, 2\}, \ i \in \{1, \dots, Q\},

\end{equation}

where $w_{k, i}$ is the provided weight for SNP i and trait k, Q is the number of variants analysed, and $p_{k, i}$ is the prior probability of SNP i for trait k. The variant-specific prior probabilities of being causal for each trait are then used to calculate the variant-specific prior probability of causality for both traits

\begin{equation}

p_{12, i} = \frac{p_{12}}{p_1 p_2} \times p_{1, i} \times p_{2, i}

\end{equation}

where p_{12}, p_1 and p_2 are as described above. This calculation of the prior probabilities is designed to ensure that the variant-specific prior probabilities satisfy two conditions: that they are proportional to $w_{k, i}$, and that they encode the same hypothesis prior probabilities as uniform priors. To see that this is the case, note that with uniform priors,

\begin{equation}

\Pr(H_k) \approx Q \times p_k, \ k \in \{1, 2\}

\end{equation}

while, with variant specific priors,

\begin{equation}

\Pr(H_k)

= \sum_{i = 1}^Q p_{k, i}

= \sum_{i = 1}^Q Q \times p_k \times \frac{w_{k, i}}{\sum_{i = 1}^Q w_{k, i}}

= Q \times p_k, \ k \in \{1, 2\}

\end{equation}

Therefore, the variant-specific priors incorporate information from the supplied variant-specific weights while not affecting the overall prior probability of the colocalisation hypotheses. When one of the priors, p_1 say, is variant-specific then we have

\begin{equation*}

\Pr(H_4)

= \sum_{i = 1}^Q p_{12, i}

= \sum_{i = 1}^Q \frac{p_{12}}{p_1 p_2} \times p_{1, i} \times p_2

= \sum_{i = 1}^Q \frac{p_{12}}{p_1} \times Q \times p_{1} \times

\frac{w_{1, i}}{\sum_{i = 1}^Q w_{1, i}}

= Q \times p_{12},

\end{equation*}

so the overall prior weight on H_4 is the same as with uniform priors. See the Supplementary Text for an introduction to the coloc method and further details about the case where both priors are variable"

Reviewer #2:

The goal of this manuscript is to improve current coloc approaches by adding weights to each variant based on heritabilities, functional annotation and eQTL-TSS weights. This is an interesting paper as coloc approaches are becoming very popular to integrate genetics of complex diseases with QTL for molecular traits.

The authors demonstrate that adding the weights (based on eQTLs, ABC, Gnocchi and PolyFun) the coloc results improve but only 2-3% in both simulated and real data. I think that adding the option "variant-specific-priors" to coloc is great, specially when people can add their own weights.

As mentioned the authors used several approaches to calculate the weights and perform the benchmarking. However there are several major assumptions in this manuscript that are not totally real and can lead to wrong inferences that need to be taken into account and also mention as limitations.

We thank the reviewer for appreciating the value of adding variant-specific prior parameters to coloc. We have now added a sentence to the results section, reproduced below, that explicitly

highlight that users of coloc will be able to supply any prior weights they choose, beyond the weights we assess in the manuscript.

"Users of coloc can supply any weights they think are informative for the causality of variants a priori, *including the methods of specifying weights we assess in this manuscript, information about variant level properties in trait relevant contexts, such as single-cell enhancer-gene maps (22), and priors that have been empirically estimated for their trait of interest, for example using PolyFun."*

The first one is that the authors mention: "This analysis showed relatively little variation between tissues in GTEx or the eQTL catalogue, suggesting that tissue-specific densities are not needed.", which I am not sure, I agree, as in Figure 1, there is not analyses by tissue. There are plenty of studies that indicate that many QTLs are tissue specific, and this may have an impact of the coloc results, if the weights are variant and tissue specific

The analysis referred to in the quoted sentence is presented in Supplementary Figure 1, (not Figure 1) which shows the distribution of eQTLs around the TSS across datasets, including different tissues in GTEx. This analysis does not suggest that eQTLs are not tissue-specific, simply that the distribution of their location relative to the TSS is not tissue specific. While responding to the comments of the other reviewer, we have now moved this sentence and the associated section to the Methods, clarifying that the sentence describes an additional analysis.

The second is the "central dogma" in molecular biology of protein and mRNA levels are correlated, which is not clearly not true. The production of a protein is clearly associated with mRNA levels. But once the protein is produced, their stability in the cell, whether is secreted, cleavage …. does not depend on the mRNA levels. There are many studies that demonstrate that protein levels in humans does not correlate with mRNA levels. The authors also say that if there is a pQTL there needs to be a eQTL is also not true. Proteogenomic studies have demonstrated that pQTL are enriched for coding variants, likely affecting intracellular trafficking, membrane affinity, cleavage, protein binding among others. Those pQTL are not eQTL and not an artifact of the antibody or aptamer binding. Also pQTL are highly tissue specific, which connect to the previous critique. Therefore, using eQTLgen or OneK1K as a weights for pQTL will lead to wrong inferences. The inferences here will be lower in this scenario as both eQTL and pQTL are from blood, but it is not clear if this will be translated well for QTL generate in other tissues.

We agree that the central dogma does not strictly imply correlation between RNA and protein levels. Various mechanisms such as nonsense mediated decay of RNA, trafficking, cleavage and protein binding of proteins as well as feedback loops from protein to RNA limit this. However, our pQTL-eQTL comparison does not require that all pQTLs should have an identifiable eQTL in the various tissues we consider, but only that some (unknown) portion of them should. Therefore, a method that detects more same gene pQTL-eQTL colocalisations, while not finding many more different gene pQTL-eQTL colocalisations, should have better performance than a method that does not. This method of benchmarking was first used in Tambets et al (2024) to benchmark different methods for colocalisation analysis, including coloc.

In addition, Sun et al (2023) recently found that 65% of pQTLs in the UK Biobank colocalised with eQTLs. It is difficult to identify reliable true positives with which to assess colocalisation approaches in real data, as most examples were themselves found using colocalisation analysis, likely biasing the assessment.

We thank the reviewer for helping us realise we were not clear in describing our motivation in these comparisons. We have now revised the text to be explicit that we do not anticipate all pQTL to have a matching eQTL, but that, given the paucity of real data true and false positive examples against which to test, we chose to examine whether including variant specific priors could alter colocalisation probabilities in an eQTL-pQTL comparison, where we expect a substantial proportion of the pairs tested to reflect true colocalisations. The relevant revised text in the manuscript is:

"Next, we sought to both evaluate the effectiveness of variant-specific priors in real world data and compare the performance of different sources of prior information. Comparing the performance of the prior-specification methods using simulations is challenging, because it is not clear how to realistically specify the true distribution of causal variant locations without using information from prior sources. Furthermore, it is difficult to reliably identify true positives with which to assess colocalisation approaches in real data, as most examples were themselves found using colocalisation analysis, likely biasing the assessment. Instead, we compare the methods on their ability to recover `ground-truth' pQTL-eQTL colocalisations, inspired by the comparison of colocalisation methods performed in (27). The rationale for this comparison is that we expect, based on the central dogma of molecular biology, that if a variant affects protein levels (i.e. is a pQTL) of a protein-coding gene then it should, in most cases, act by affecting the gene's expression (i.e. is an eQTL). Therefore, an unknown but large proportion of detectable cis-pQTLs should have a colocalising eQTL signal, provided that eQTLs and pQTL are tested in similar tissues and contexts. Indeed, recently 65\% of pQTLs called in the UK Biobank were found to colocalise with eQTLs (28). Methods that detect more same gene pQTL-eQTL colocalisations, while not finding many more different gene pQTL-eQTL colocalisations, should have better performance than methods that do not."

The other major limitation, not mentioned or discussed on the manuscript is that all the databases uses here are based on Non-Hispanic white datasets (I think OneK1K has some non-white datasets, but not clear how many and how was used). Different populations have different LD and different QTLs. Leverage other LD maps may have more impact on improving coloc results that incorporating weights. At least a discussion of this should be included on the manuscript.

We agree that likely a better way to improve fine mapping and therefore colocalisation performance is to use data from multiple ancestries. Indeed, it is often said that colocalisation requires that both datasets have equal LD, but that is not true for the single-coloc approach, which makes no assumptions about LD in its mathematical derivation, nor the coloc with susie approach, where the LD can be different in the different traits. We also agree the general lack of QTL datasets in non-white individuals is a limitation of the paper and reflects the current QTL data landscape. We have added a paragraph about ancestry in the discussion, which discusses

the need for more multi-ancestry colocalisation studies, notes that colocalisation can be performed cross-ancestry, and acknowledges his limitation of the focus on white European ancestry in our work. The relevant text is:

"... all QTL datasets in this study are from primarily European-ancestry cohorts, reflecting a general bias in currently available QTL data. The use of data from non-European-ancestry individuals may improve the performance of colocalisation analysis, especially for GWAS performed in individuals of diverse ancestry. We note that coloc does not require both datasets to have the same LD structure so it can be used to perform cross-ancestry colocalisation analyses."

Finally, we have updated the title of the manuscript to "Variant-specific priors clarify colocalization analysis" to better reflect the positive impact of variant-specific priors. In addition, have made several small changes to the text to fix newly identified errors or imprecisions. We have also now included Supplementary Figure 4 in Figure 3, which we feel makes the manuscript easier to read. The updated Figure 3 is reproduced below:

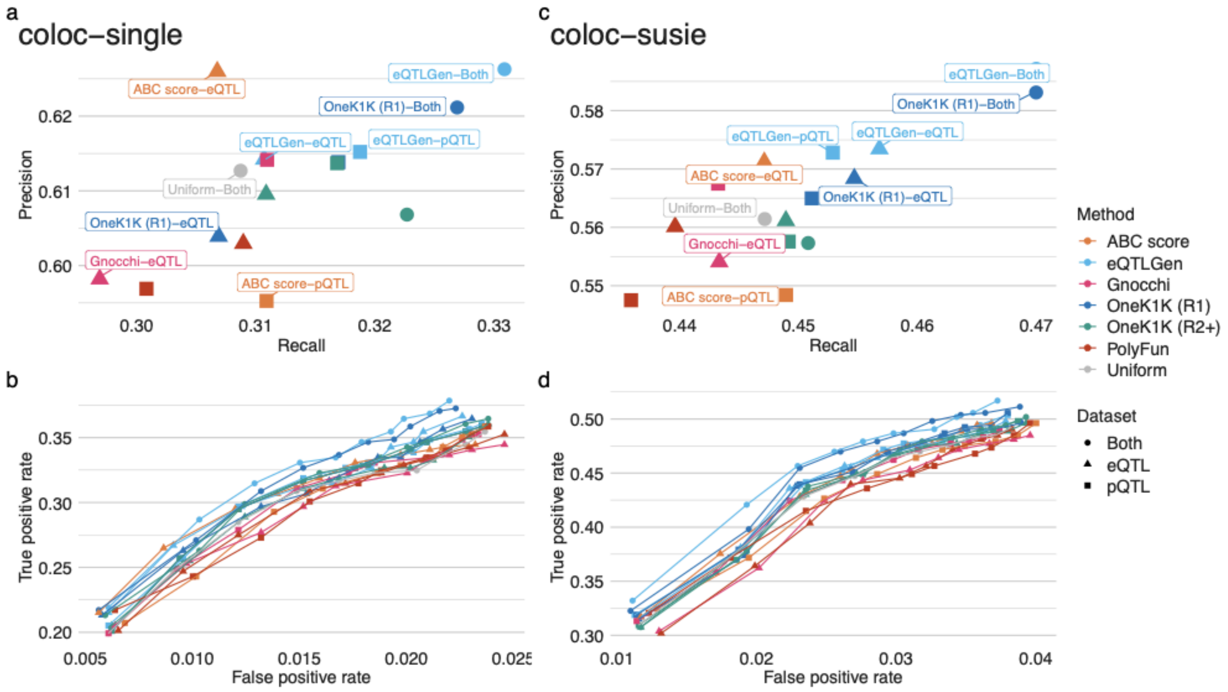

Figure 3: pQTL-eQTL colocalisation performance assessment for coloc with a single causal variant assumption. *a) Recall and precision of colocalisation at the $\Pr(H_4) > 0.8$ significance threshold based on ground-truth pQTL-eQTL colocalisations. All 6 different sources of prior information were applied to either the eQTL or pQTL data, with the 3 eQTL-TSS distance density priors additionally applied to both datasets. b) A ROC-style analysis of the ground-truth pQTL-eQTL data using the $\Pr(H_4)$ thresholds: $0.5, 0.55, \dots, 0.95$. In both*

panels coloc assuming a single causal variant (coloc-single) is used. c) As in a) but using coloc with SuSiE (coloc-susie). d) As in b) but using coloc with SuSiE (coloc-susie).
