# Supplementary material for: Variant-specific priors clarify colocalisation analysis
Source: PLoS Genet. 2025 May 27;21(5):e1011697. doi: 10.1371/journal.pgen.1011697 (PMC12140431; doi:10.1371/journal.pgen.1011697)
Supplement: Attachment — Submitted filename: vsp-reviewer-response_auresp_2.pdf [file pgen.1011697.s012.pdf]

Reviewer #2: This is a revised manuscript focused on improving coloc approaches by adding variant specific weights.
My previous concerns were related to having weights based only on eQTL and using only EUR-specific weights. The authors have addressed my comments related with the European-specific weights.
I still do fully not agree with their justification for the pQTL. The authors indeed indicate that only 65% of the pQTL colocalize with eQTL, so a large number of then, 35% do not. And most of this pQTLs that do not colocalize are due to posttranslational events. I think that talking here about the central dogma, is indeed, simplifying biology too much that is misleading. Proteins levels are regulated at many more levels than mRNA, and proteins, a and not RNA, are normally the effector molecules in diseases and biological traits. Saying that mRNA -related data can just be translated into protein data is one of the reasons so many loci are still no solved. I would suggest removing the wording related the central dogma and that the pQTLs are expected to be eQTL as this is clearly erroneous and misleading.

We have now omitted the text linking the pQTL-eQTL comparison to the central dogma. The **removed** text is reproduced below.

"
The rationale for this comparison is that we expect, based on the central dogma of molecular biology, that if a variant affects protein levels (i.e. is a pQTL) of a protein-coding gene then it should, in most cases, act by affecting the gene's expression (i.e. is an eQTL). Therefore, an unknown but large proportion of detectable cis-pQTLs should have a colocalising eQTL signal, provided that eQTLs and pQTL are tested in similar tissues and contexts. Indeed, recently 65\% of pQTLs called in the UK Biobank were found to colocalised with eQTLs (Sun 2023)
"

It would be ok with me, if the authors just have a few sentences in the discussion that including tissue-specific and other molecular traits QTL can improve the coloc. The can add it to the sentence where they mention the users can include their own weight.

We agree that this point is worth making explicitly and have now included the following text in the discussion section of the manuscript.

"
Finally, we note that using variant-specific prior probabilities is not expected to replace the need to perform colocalisation using a wider range of tissue- and context-specific eQTLs and more diverse molecular trait QTLs.
"

In addition, we have updated the text of the paper to clarify that variant-specific priors have now been implemented in v6.0.0 of coloc.
